# Early-stage fusion of EEG and fNIRS improves classification of motor imagery

**DOI:** 10.3389/fnins.2022.1062889

**Published:** 2023-01-09

**Authors:** Yang Li, Xin Zhang, Dong Ming

**Affiliations:** ^1^Academy of Medical Engineering and Translational Medicine, Tianjin University, Tianjin, China; ^2^The Laboratory of Neural Engineering and Rehabilitation, Department of Biomedical Engineering, School of Precision Instruments and Optoelectronics Engineering, Tianjin University, Tianjin, China; ^3^The Tianjin International Joint Research Center for Neural Engineering, Academy of Medical Engineering and Translational Medicine, Tianjin University, Tianjin, China

**Keywords:** EEG, fNIRS, hybrid-BCI, modality fusion, motor imagery

## Abstract

**Introduction:**

Many research papers have reported successful implementation of hybrid brain-computer interfaces by complementarily combining EEG and fNIRS, to improve classification performance. However, modality or feature fusion of EEG and fNIRS was usually designed for specific user cases, which were generally customized and hard to be generalized. How to effectively utilize information from the two modalities was still unclear.

**Methods:**

In this paper, we conducted a study to investigate the stage of bi-modal fusion based on EEG and fNIRS. A Y-shaped neural network was proposed and evaluated on an open dataset, which fuses the bimodal information in different stages.

**Results:**

The results suggests that the early-stage fusion of EEG and fNIRS have significantly higher performance compared to middle-stage and late-stage fusion network configuration (*N* = 57, *P* < 0.05). With the proposed framework, the average accuracy of 29 participants reaches 76.21% in the left-or-right hand motor imagery task in leave-one-out cross-validation, using bi-modal data as network inputs respectively, which is in the same level as the state-of-the-art hybrid BCI methods based on EEG and fNIRS data.

## 1. Introduction

Brain–computer interfaces (BCIs) are communication systems that utilize control signals generated by the brain to interact with the surrounding environment without the participation of the peripheral nervous system and muscles (Nicolas-Alonso and Gomez-Gil, 2012). These years have witnessed thriving progress in the field of BCI. Motor imagery (MI) is one of the common paradigms in BCI research (Kaiser et al., 2011), which is accomplished by imagining performing the given task (Jeannerod, 1995), such as grabbing (Herath and Mel, 2021), lifting (Kasemsumran and Boonchieng, 2019), and so on. MI-BCIs are widely used to aid patients with motor function impairments caused by stroke (Ang et al., 2010), amyotrophic lateral sclerosis (Lulé et al., 2007), spinal cord injury (Cramer et al., 2007), and so on, either for daily-life assistance or rehabilitative training. Since motor imagery tasks induce event-related desynchronization and synchronization (ERD/ERS) in EEG (Jeon et al., 2011), various feature extraction algorithms have been designed to detect ERD/ERS activities in EEG (Kee et al., 2017; Selim et al., 2018; Sadiq et al., 2019; Dagdevir and Tokmakci, 2021). However, due to its nonstationary nature, EEG is considered as bio-signals of extremely low signal-to-noise ratio with spatial ambiguity and distortion (Hallez et al., 2007). EEG feature extraction process, which is highly dependent on prior knowledge, is challenged by its high time complexity, imposing the risk of information loss (Zhang et al., 2018, 2021). Many researchers turned to deep learning methods for EEG feature extraction. For example, Schirrmeister et al. (2017) proposed an end-to-end learning network called ConvNets that was able to learn the spectral power modulation of different frequency bands and produce accurate spatial mapping for learned features. Lawhern et al. (2018) proposed a compact convolutional neural network to accurately decode EEG recorded from various paradigms.

The low spatial resolution characteristic of EEG leads to challenges in the accurate localization of cortical activation sources despite the fact that EEG signals are the most widely used bio-signals in BCIs (Liu et al., 2021). Due to its disadvantage in spatial resolution, some researchers attempted to incorporate the information from functional near-infrared spectroscopy (fNIRS) data to improve the performance of BCIs (Pfurtscheller, 2010; Fazli et al., 2012; Buccino et al., 2016). fNIRS measures oxygenated and deoxygenated hemoglobin (HbO and HbR) using near-infrared light (Fazli et al., 2012). On the one hand, the fusion of EEG and fNIRS has technical support because the electrophysiological signal and the inner edge light signal are not affecting each other. On the other hand, fNIRS-based BCIs are most commonly of the active type, where users react purposefully and independently (Khan and Hong, 2017). Therefore, plenty of mental tasks exploit fNIRS signals to assess brain status, which have proven to be effective in previous studies (Hong et al., 2015). Yin et al. introduced joint mutual information (JMI) to combine features and optimize BCIs, which was used to classify MI tasks with different strengths and speeds when clenching a fist. JMI reached an accuracy of 89 ± 2% with 1–5% improvement compared to using EEG or fNIRS alone. Al-Shargie et al. applied canonical correlation analysis to decode EEG-fNIRS and maximized the correlation between EEG and fNIRS to classify the influence of psychological stress on the prefrontal cortex (Al-Shargie et al., 2017). Sun et al. used tensor fusion and *p-*order polynomial fusion with deep learning technologies, which improved the accuracy at the cost of increased computational complexity and reduced the stability (Sun et al., 2020).

There are relatively mature methods and a relatively clear consensus for dealing with multimodal fusion problems in the field of computer vision. Depending on those methods, the researchers combined features in the early or late stage to achieve the best results. For example, Aygün et al. (2018) adapted various fusion methods, which were previously used in video recognition problems, to solve the brain tumor segmentation problem and conducted the related experiments in the BRATS dataset in the early, middle, and late fusion methods. A Y-shape network is widely used in tasks with multimodal inputs. The multimodal models usually have their own encoders on each modality. For example, the image encoder and the language encoder form a twin tower structure model that is used for loss calculation in CLIP, which is a training structure of language–image multimodal fusion (Radford et al., 2021). Lan et al. (2019) used a Y-shaped network to combine two encoders with the path of one decoder and extract more information from raw data. As a result, the Y-shape network is extremely helpful for data reconstruction and multimodal fusion. However, in the field of biomedical signal processing, there is no consensus on the processing of physiological signals from different modalities. Fusion of EEG and fNIRS information is conducted mostly arbitrarily at the feature level, which has been proven to be suitable for several specific user cases. When and how to effectively combine the bimodality data is still unclear. This study conducted experiments on an open dataset. A compact Y-shaped ANN architecture has been proposed and validated to investigate the EEG-fNIRS fusion methods and strategies. The main framework of EEGNet is used in the EEG processing branch, which is a proven successful framework for EEG data analysis. As the temporal resolution of fNIRS is low and minimal frequency information is present, only the second and third modules of EEGNet are used in the fNIRS processing branch. The results suggest that neural networks with EEG-fNIRS features integrated at an early stage demonstrated statistically higher accuracy. The final classification accuracy of the proposed method reaches 76.21%, which is at the same level compared to the state-of-the-art on the investigated open dataset in discriminating left and right motor imagery.

This article is organized as follows. In the “Materials and methods” section, the dataset is briefly introduced, and the preprocessing method and the proposed framework are demonstrated in detail. In the “Results” section, the results are presented. In the “Discussion” section, an in-depth discussion is presented. In the “Conclusion” section, conclusions are presented.

## 2. Materials and methods

### 2.1. Datasets

Shin et al. released two publicly available datasets of EEG-fNIRS multimodal, which were Dataset A, left-hand motor imagery and right-hand motor imagery, and Dataset B, mental arithmetic and relax imagery (Shin et al., 2017). The primary focus of this study was MI classification, and Dataset A was used to conduct a series of experiments and analyze further in this study.

For Dataset A, there were 29 participants (14 men and 15 women), all of whom had minimal experience with motor imagery experiments. In the experiment, a black arrow pointing to the left or right was shown in the middle of the screen for the first 2 s. Then, the arrow disappeared and a fixed black cross was shown on the screen for 10 s. All the participants were instructed to perform kinesthetic motor imagery at a speed of approximately 1 repetition per s, such as imagining a designated hand opening and closing as if they were grasping a ball, followed by a rest period of 10–12 s. Finally, there were 30 trials for each task of each participant. Common spatial pattern (CSP) features of EEG data and the mean and slope values of fNIRS signals were extracted from the data as features. A sliding window was used to conduct 10 × 5-fold cross-validation on the unimodal data and bimodal data, respectively, with window size set to 3 s, step size set to 1 s, and the range of sliding window set to between 5 s before the cue and 20 s after the cue. sLDA was used as a classifier to classify data between left and right motor imagery tasks.

In their article, the average classification accuracy of the 10 × 5-fold cross-validation under each window was considered as the classification accuracy of this window. In addition, the maximum classification accuracy among all the windows was regarded as the final classification accuracy for each participant. The highest classification accuracy of EEG-only was about 65%, and the highest classification accuracy of unimodal classification was HbO-fNIRS, which was approximately 57% according to the resulting figure.

### 2.2. Pre-processing

**Figure 1:**
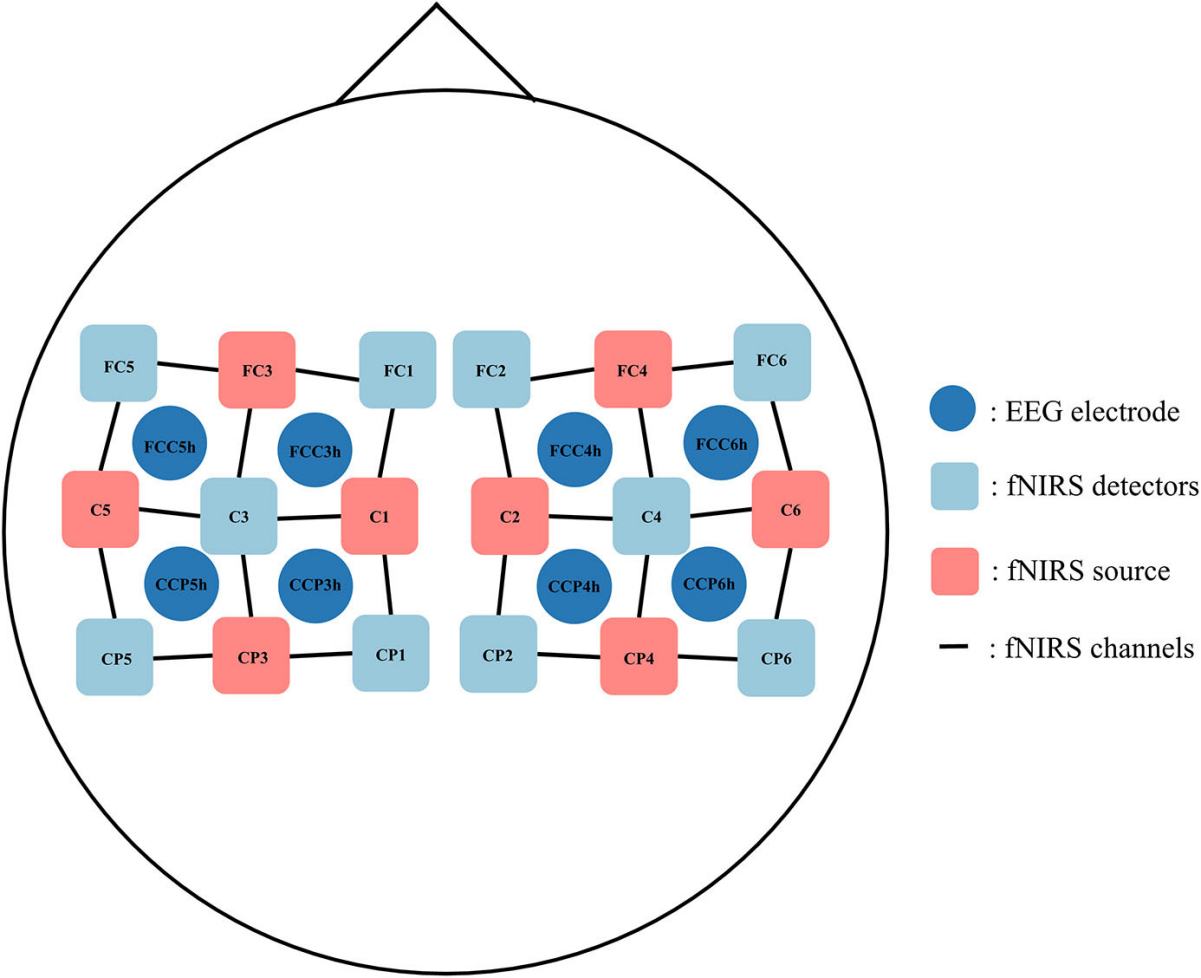
The placement of EEG electrodes, fNIRS sources, and fNIRS detectors.

For dataset A, the EEG was recorded using a BrainAmp EEG amplifier, with the sampling rate set to 200 Hz in the original dataset. First, the data were downsampled from 200 to 128 Hz, and the channels related to EOG were removed for later analysis. Then, the EEG data were re-referenced to the common average reference. A band-pass filter with a frequency range of 8–25 Hz was applied to remove noise, leaving the μ-band and low-β band data unmodified. Since we wanted to focus on channels related to the sensorimotor cortex and maintain the correspondence with fNIRS optical channels, eight relevant electrodes were chosen around the sensorimotor cortex, namely, FCC5 h, FCC3 h, CCP5 h, CCP3 h, FCC4 h, FCC6 h, CCP4 h, and CCP6 h (shown in Figure 1). The amplitude of the signals was normalized to [−1, 1] for subsequent processing.

The sampling rate of the fNIRS signal was set to 10 Hz in the original dataset. First, the data were up-sampled from 10 Hz to 128 Hz to be consistent with EEG data (Abtahi et al., 2020). We chose eight optical channels (6 emitters and 6 detectors with 3-cm optrode separation) around the sensorimotor cortex, i.e., FC3-FC5, FC3-FC1, C5-C3, C1-C3, FC4-FC2, FC4-FC6, C2-C4, and C6-C4 (shown in Figure 1), whose positions corresponded to the selected EEG channel locations, to ensure spatial consistency of the recorded data. The modified Beer–Lambert law was used to convert the raw light intensity data to the relative oxyhemoglobin and deoxyhemoglobin concentrations. Then, a band-pass filter with a frequency range of 0.01–0.1 Hz was used to remove the effect of physiological noises such as heartbeat, breath, and other artifacts. We extracted 10 s data during the task period, and data from 5 s to 2 s before the visual cue were used to remove the baseline. Finally, the amplitude of the signals was normalized to [−1, 1] for subsequent processing.

### 2.3. Fusion network

The basic network structure is inspired by the EEGNet (Lawhern et al., 2018). The original EEGNet is composed of three modules. The first module is a temporal-domain convolution layer through which the time-frequency features of the signals are constructed. The kernel size is set to (1, *fs*//2), where *fs* is the sample rate of signals, and the sign // denotes the rounding operation. The second module is depth-wise convolution through which spatial filters are generated and more task-related channels are selected by the convolution kernel, where the kernel size is set to (*N*_*chan*_, 1), where *N*_*chan*_ denotes the number of EEG channels. Average pooling is used to down-sample the feature dimension. The third module is a separable convolution layer, which consists of depth-wise convolution and pointwise convolution.

**Figure 2:**
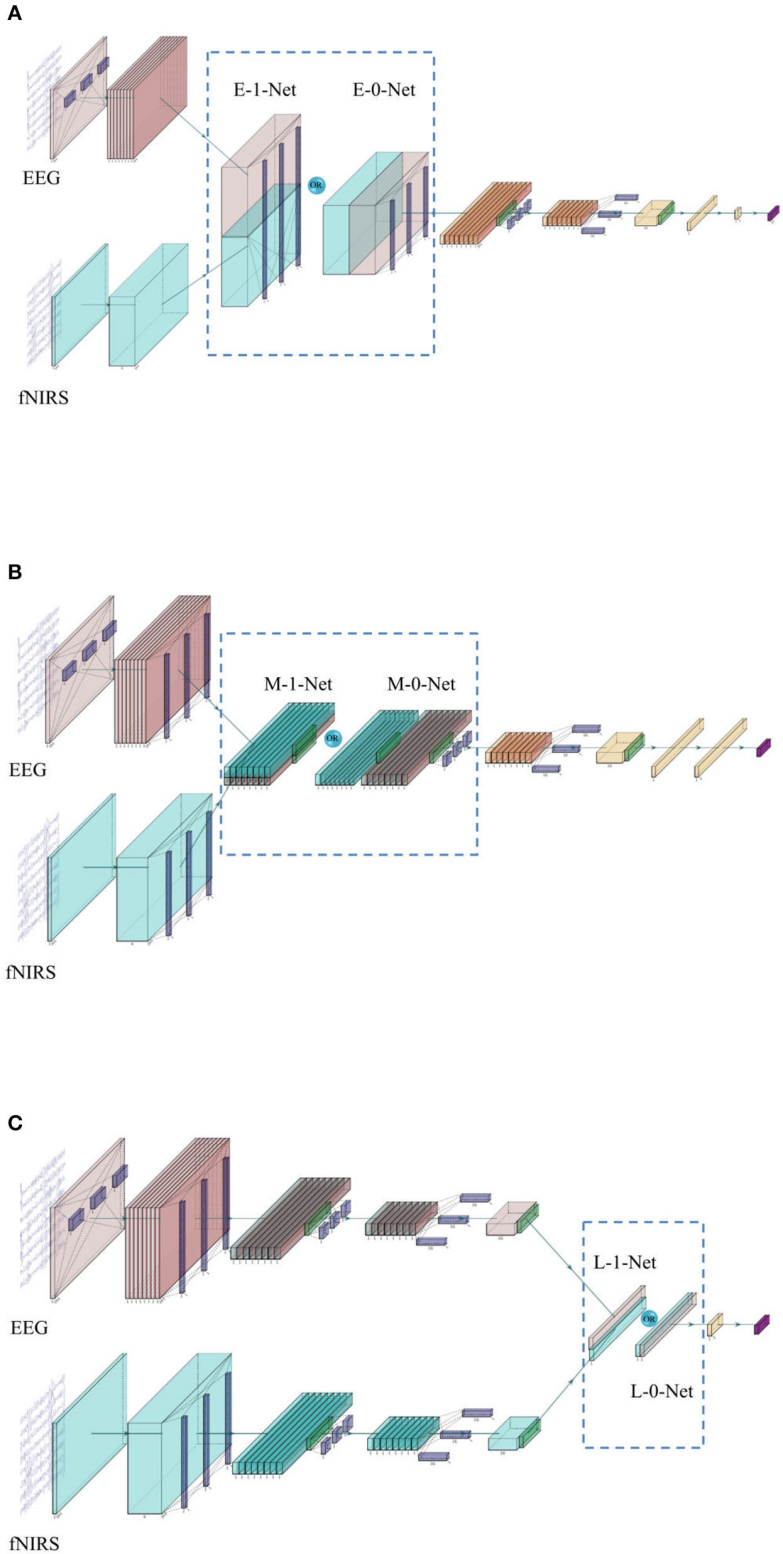
**(A)** The network architecture of early stage fusion, which is referred to as E-0-Net and E-1-Net in the following contents, depends on whether the concatenation was performed on the 1st or 2nd dimension. **(B)** The network architecture of middle-stage fusion, which is referred to as M-1-Net and M-0-Net in the following contents. **(C)** The network architecture of late-stage fusion, which is referred to as L-1-Net and L-0-Net in the following content.

In this study, we followed EEGNet architecture with three complete modules for EEG data. For fNIRS, we only used the second and third modules since the fNIRS signals did not contain much information in the frequency domain due to the low sampling rate, and the features are mostly extracted from the time domain. At the end of the Y-shaped network, a SoftMax layer was used as a classifier to generate the outputs. The complete network architecture is shown in Figure 2.

In the literature, one of the commonly used fusion methods is the concatenation of features from each modality (Baltrusaitis et al., 2019). The network architecture is shown in Figure 2. In this study, three similar networks are proposed to investigate the effect of fusing bimodal features in different stages, i.e., before depth-wise convolution (referred to as E_0_Net and E_1_Net, please see Figure 2A), before separable convolution (referred to as M_0_Net and M_1_Net, please see Figure 2B), and before flatten layer (referred to as L_0_Net and L_1_Net, please see Figure 2C), where E, M, and L represent early stage fusion, middle-stage fusion, and late-stage fusion, respectively, and numbers 0 or 1 represent concatenation fusion performed at the depth-dimension or the channel-dimension. In this study, to maintain the integrity of the original design of EEGNet, the proposed network used the same hyperparameters as proposed in the original EEGNet paper (Lawhern et al., 2018); only the kernel size of the first layers in EEG branch was tuned as we had a very limited number of trials in the investigated open dataset and a larger kernel size created more parameters to be learned. We tried kernel sizes of (1, 34), (1, 44), (1, 54), and (1, 64) to perform temporal-domain convolution on EEG data to have the best model performance during the training process.

Table 1 summarizes the number of network parameters with different fusion strategies. The numbers of neural network parameters for different fusion methods are similar, except for M_0_Net. For the open dataset used in this study, the amount of data from a single participant in one particular type of task is too small when using networks with a large number of parameters. Therefore, the use of a lightweight network can alleviate overfitting to a certain extent.

**Table 1:** The number of all parameters.

**Method**	**Number of parameters**
E_0_Net	3,792
E_1_Net	3,792
M_0_Net	8,880
M_1_Net	4,172
L_0_Net	4,176
L_1_Net	4,176

### 2.4. Model training

Early stopping is a form of regularization that prevents overfitting by stopping the iteration number. When training error decreases quickly, we hope that the model continues to be trained and that the generalization losses have a higher chance of being “repaired”. In this study, we used an early stopping criterion that assumes that overfitting does not begin until the error decreases slowly. The algorithm is shown in Equations (1) and (2), referred to from Prechelt (2012). In this study, we did not use a validation dataset and we used an early stopping strategy to reduce jitter.


(1) $$P_{k}=\left(t\right)1000\cdot (\frac{\sum_{t^{\prime }=t-k+1}^{t}E_{tr}\left(t^{\prime }\right)}{k\cdot \min_{t^{\prime }=t-k+1}^{t}E_{tr}\left(t^{\prime }\right)}-1)$$

(2) $$\begin{array}{l} P_{k}\left(t\right)<\alpha \end{array}$$

where *k* is the training strip, *Etr* is the training error, and α is the threshold value. When *Pk*(*t*) is less than α, we think that it is time to stop. In this study, *k* is 10 and α is 0.001.

Due to the limitation in the amount of data, for each participant, there were only 30 trials for each motor imagery task in the open dataset. A data augmentation method designed for long-interval EEG-fNIRS hybrid BCI applications was used to expand the size of the dataset. Due to the limitation in response time of fNIRS signals, the time intervals between experiment tasks were more than 10 s. Therefore, data augmentation can be achieved by repetitively sampling sub-trials from a single trial.

In this study, two training strategies were adopted. For training Strategy A, the window size was set to a 3-s time window, and the step size was set to 3 s. Then, each 10-s trial was divided into 3 sub-trials without overlapping. Therefore, the number of trials for each participant from one task was expanded to 90 trials and was used for neural network training. All the sub-trials were randomly shuffled before the train-test segmentation of data. The data were then randomly divided into an 80% training set and a 20% testing set. The proposed neural networks either were trained for 500 epochs or met the early stopping criteria.

For training Strategy B, we used leave-one-out cross-validation for each participant. The voting method was used to train the network with the idea of decision fusion. We divided each trial with a window size of 3 s and a step size of 1 s. Each trial was divided into 8 sub-trials. In the training set and testing set, two overlapping sub-trials from the same trial did not appear at the same time. The data from the open dataset were further expanded without the training set leakage. During the training process, all data were randomly shuffled. The proposed neural networks were either trained for 500 iterations or met the early stopping criteria.

### 2.5. Voting mechanism

Ensemble learning is one of the most popular research topics (Wozniak et al., 2014). It extracts a set of features through a diversity of projections on data using multiple machine learning algorithms and performs various transformations of features. Then, various classification algorithms are used to generate prediction results based on the extracted features. Information from the abovementioned results is integrated to achieve better performances than information obtained from any stand-alone algorithm (Dong et al., 2020). For classification tasks, the voting method is often used to improve the final results (Zhou, 2012). One of the commonly used voting combinations is the majority voting combination, where the predicted results of most are considered as the final output. The voting algorithm is shown in Equation (3).


(3) $$\hat{y}=\{\begin{array}{cc} 1,\; & n_{\hat{yi}}>n_{\hat{y0}}\\ 0,\; & n_{\hat{yi}}<n_{\hat{y0}} \end{array}$$

where $n_{\hat{yi}}$ is the number of test samples with its predicted results being 1. $n_{\hat{y0}}$ is the number of test samples with its predicted results being 0, and ŷ is the final predicted result of this trial.

In this study, we used a sliding window of 3 s with a step size of 1 s. Therefore, each trial is divided into 8 sub-trials. Then, a leave-one-out cross-validation scheme was used to test the model performance for each actual trial after data augmentation from each participant. The predicted results of the majority voting combination of 8 sub-trials were the final prediction results of one trial.

## 3. Results

### 3.1. Data augmentation

Deep convolutional neural networks have achieved outstanding performance in many areas, which is driven by improvements both in computational power and the availability of large datasets. However, it is extremely difficult to acquire or collect large datasets for lots of application fields, such as datasets of physiological signals. If a small dataset was used to train a model with a large number of parameters, overfitting would happen, resulting in poor generalization performance. In the related studies on computer vision, overfitting can be alleviated by data augmentation, such as geometric transformation, random cropping, feature space manipulation, adversarial training, and so on, to improve the model performance and expand its limited dataset (Shorten and Khoshgoftaar, 2019).

In the dataset investigated in this study, there were only 30 trials in each task for each participant in this open dataset, and each trial is 10 s long. Data augmentation was used in the model generation to improve the model performance. The data from one trial of 10 s were truncated to three trials as 0–3 s, 3–6 s, and 6–9 s without overlap. Through the augmentation process, the original dataset was expanded to three times its original size.

**Figure 3:**
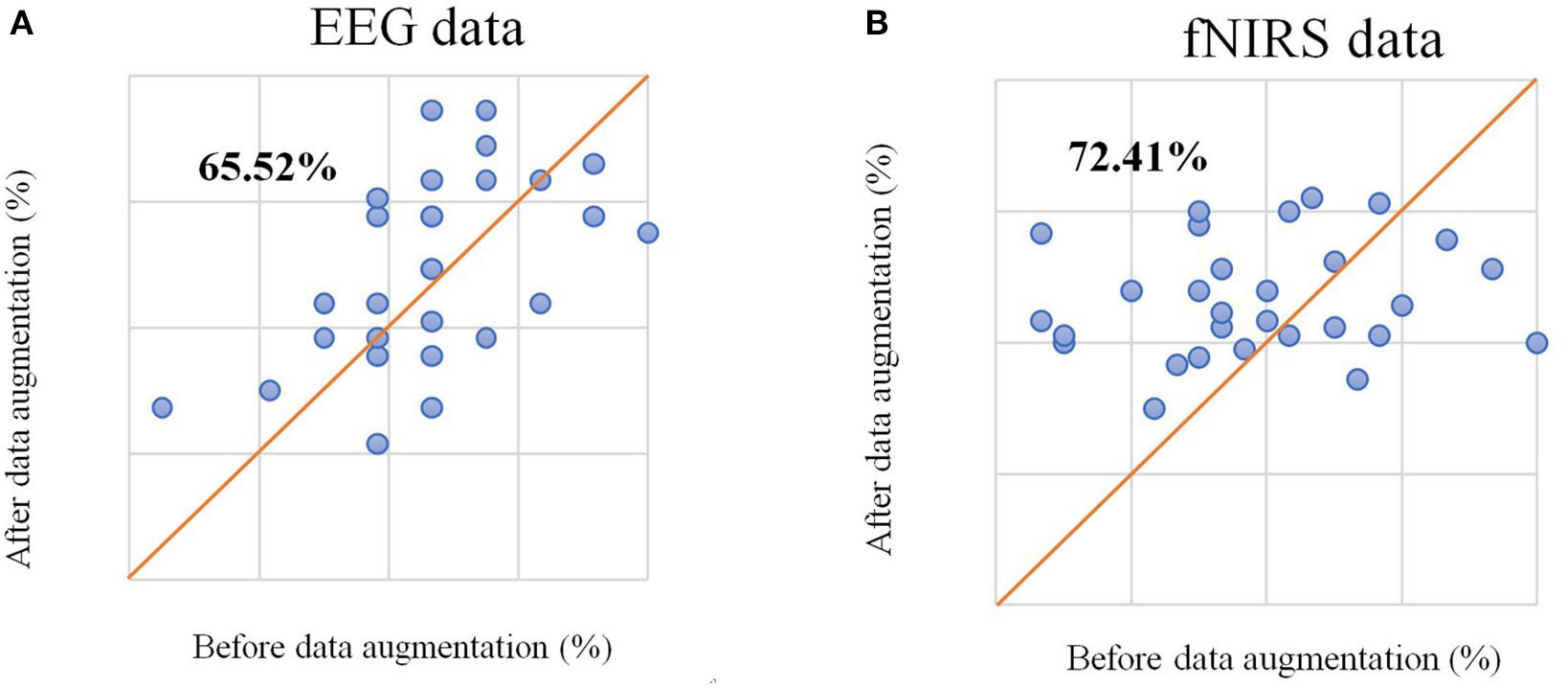
**(A)** The scatter plot of classification accuracy of each participant based on EEG signals before and after data augmentation. **(B)** The scatter plot of classification accuracy of each participant based on the fNIRS signal before and after data augmentation.

We selected unimodal data (EEG-only and HbO-only) without data augmentation from different time windows using the method from the original dataset study (Shin et al., 2017) and chose the average accuracy of all participants among different time windows as average accuracy for statistical analysis. The CSP algorithm was used to extract features from augmented EEG data, the mean and slope features were extracted from HbO-fNIRS, and sLDA was used as a classifier to generate a classification model. The highest classification accuracy of left-right motor imagery classification with only EEG data (referred to as EEG-only in the following content) was 66.09%, and the highest classification accuracy of left-right motor imagery classification with only HbO data (referred to as fNRIS-only in the following content) was 54.31%, which were similar to the results of the original study. After data augmentation, the highest average accuracy for EEG-only reached 69.25% and for fNIRS-only reached 58.33%. The average accuracy improved for both EEG-only and fNIRS-only. It can be seen from Figure 3 that the classification accuracy of 65.52% of participants improved for EEG and that 72.41% of participants improved for fNIRS compared with the original data. This method of data augmentation not only expands the dataset but also improves the classification performance.

**Figure 4:**
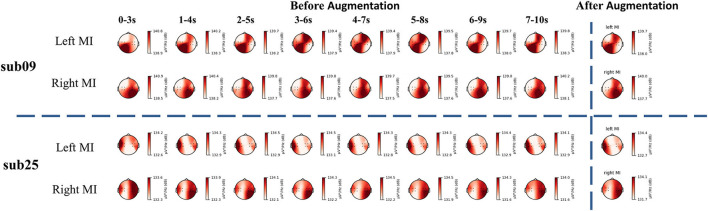
The PSD analysis of EEG signal. The PSD analysis of sub09 is shown above the horizontal dashed line and that of sub25 is shown below the horizontal dashed line.

Similarly, we used artificial neural networks to classify EEG and fNIRS data from different tasks. The average accuracy of all participants is 65.00%. Sub01, sub09, sub16, sub25, sub26, and sub27 demonstrated good classification performance using EEG data with a classification accuracy of more than 80%. Participants with top model classification performance (sub09 and sub25) were analyzed with power spectral density (PSD) (shown in Figure 4). Clear EEG power lateralization was identified both before data augmentation and after data augmentation. The proposed method of data augmentation can maintain the original temporal-spatial characteristic in the EEG data.

**Figure 5:**
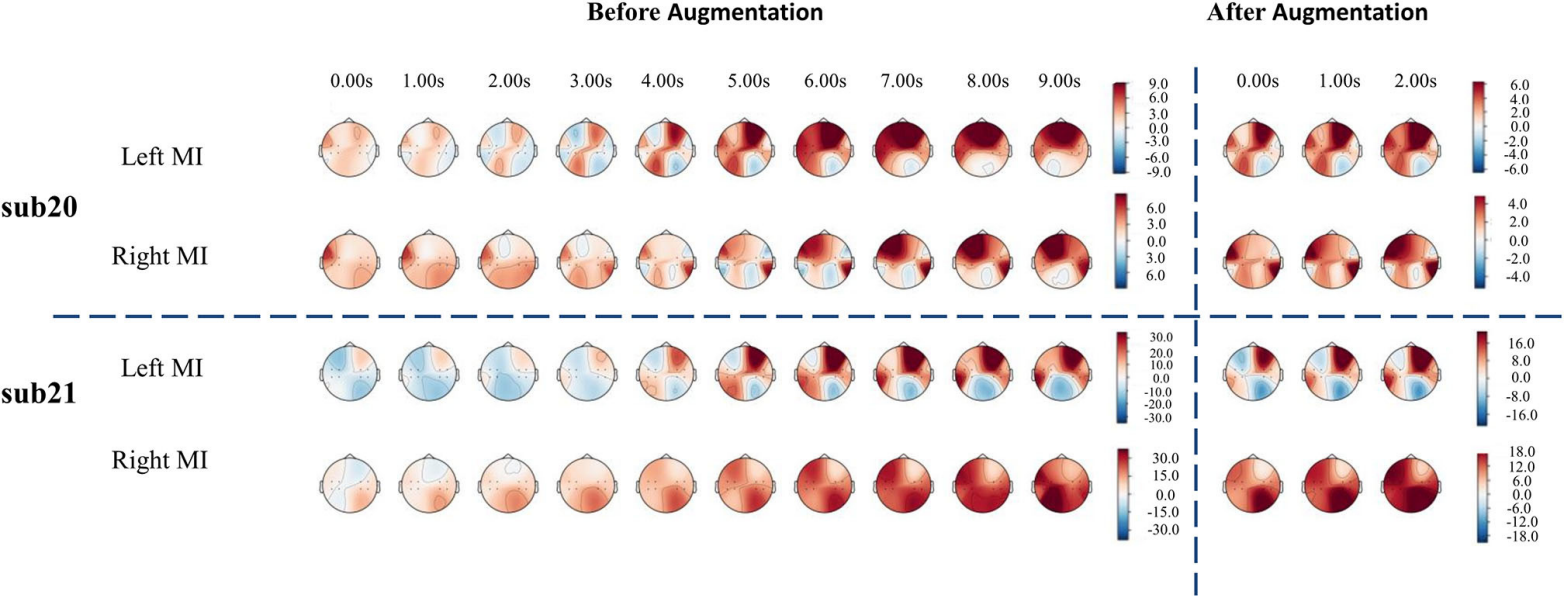
The temporal-domain analysis of the fNIRS signal. The temporal-domain analysis of sub20 is above the horizontal dashed line, and that of sub21 is below the horizontal dashed line.

For fNIRS-HbO, the average accuracy of the lightweight network of all participants is 63.13%. Sub09, sub19, sub20, sub21, sub24, and sub29 were able to demonstrate good classification performance using fNIRS, with the classification accuracy reaching more than 70%. We used the cerebral oxygen exchange (COE, where COE value = HbO – HbR) (Naseer and Hong, 2015) as input and selected the participants with the top performances (sub20 and sub21) for temporal-domain analysis. As shown in Figure 5, before data augmentation, clear lateralization of the COE values can be identified in both left-hand and right-hand motor imagery tasks: the COE values of the left channels were significantly higher than that of the right channels during the left-hand motor imagery, and COE values of the right channels were significantly higher than that of the left channels during the right-hand motor imagery, which was consistent with the results presented in the literature (Asahi et al., 2004; Hétu et al., 2013). At the same time, data augmentation with a sliding window of 3 s with a 1 s step size also demonstrated similar lateralization characteristics, as shown in Figure 5. The proposed data augmentation method did not disturb the temporal-spatial characteristics of original fNIRS data and maintained good consistency.

### 3.2. Model generation

**Figure 6:**
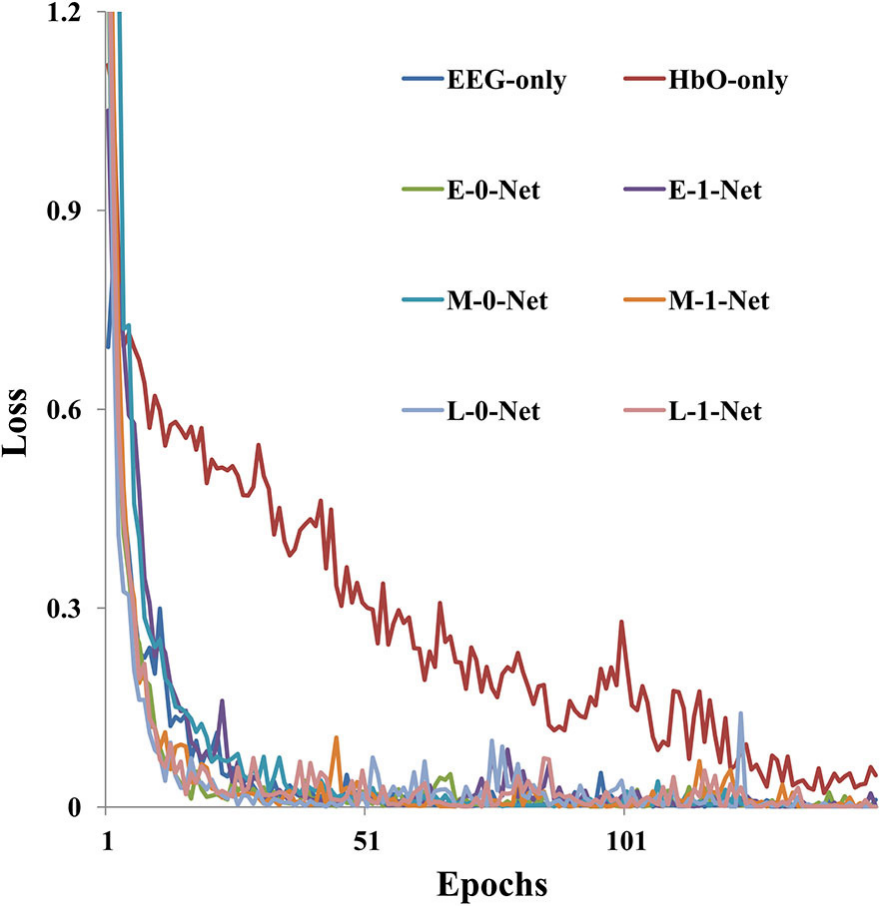
The model training loss (sub09 as an example) varies with the number of epochs under different network architectures.

As shown in Figure 6, model training loss varied with the number of iterations under different network architectures. It was clear that the value of training loss reduced as the number of epochs increased. In addition, the convergence speed was the lowest when fNIRS-only data were used for model generation, which required 150 epochs before convergence. However, for EEG-only data, the convergence speed was faster than fNIRS-only data, and the training reached convergence within 50 epochs. The model converged faster with a bimodal fusion network than with a single-modality network.

### 3.3. Test results

**Figure 7:**
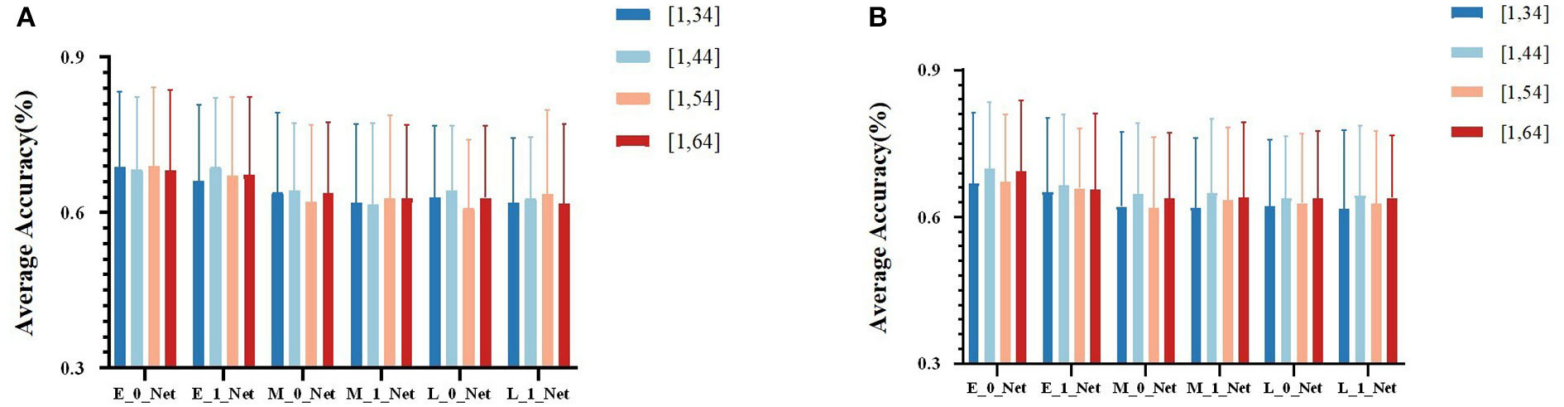
**(A)** The average classification accuracy of all participants using EEG-HbO in different fusion strategies. **(B)** The average classification accuracy of all participants using EEG-HbR in different fusion strategies.

Due to the obvious temporal characteristic difference between EEG and fNIRS, we attempted different sizes of kernels (parameters used for temporal-domain convolution) during the comparison of fusion results at different stages. We divided the results into four groups at different stages, namely, (1, 34), (1, 44), (1, 54), and (1, 64). In Table 2 for different kernel sizes, the classification accuracies of the two methods of early stage fusion were significantly higher than that of other fusion methods. In addition, the accuracies of the two early fusion methods were both within the range of 69–70%. For middle-stage fusion methods, the classification accuracies ranged from 65 to 66%. For late-stage fusion methods, the classification accuracies ranged from 62 to 63% (see Figure 7). Since none of these results conformed to a normal distribution, the Wilcoxon signed-rank test was adopted to investigate the statistical significance. Table 2 summarizes the results of the significance analysis of different fusion methods. We observed that *p-*values between early stage fusion and middle-stage fusion or for late-stage fusion were all below 0.05 regardless of the kernel size, which represents the statistical significance of the performance difference between the early stage fusion method and other stage fusion methods. The performance of early stage fusion was significantly higher than late-stage fusion. We also optimized the proposed bimodal fusion network to achieve the best classification performance further. We further optimized the size of the pooling layer and the number of convolution filters to optimize the model performance. For the pooling layer, we searched from (1, 4) to (1, 16), with (1, 4) as the step size and four options in total. Other hyperparameters were still the same as in the original paper of EEGNet. The optimal parameters are shown in Table 3. In addition, the optimal average accuracy was 71.60% and the standard deviation was 1.42% using EEG-HbO with E-N-0. The optimal average accuracy was 71.21%, and the variance was 1.88% using EEG-HbR with E-N-0.

**Table 2:** Statistical analysis results between the different fusion methods.

**HbO**	**Kernel size**	**Early-mid (*N =* 57)**	**Early-late (*N =* 57)**	**Mid-late (*N =* 57)**	**Dim_0-Dim_1 [E-M] (*N =* 28)**
*P*-values	(1,34)	0.0007	0.0011	0.3550	0.0566
	(1,44)	0.0003	0.0013	0.7033	0.2801
	(1,54)	0.0007	0.0007	0.5872	0.2864
	(1,64)	2.8884	0.0001	0.2066	0.2594
**HbR**	**Kernel size**	**Early-mid (*****N** =* **57)**	**Early-late (*****N** =* **57)**	**Mid-late (*****N** =* **57)**	**Dim_0-Dim_1 [E-M] (*****N** =* **28)**
*P*-values	(1,34)	0.0024	0.0014	0.5217	0.0540
	(1,44)	0.0033	0.0045	0.3226	0.0257
	(1,54)	0.0025	0.0034	0.6294	0.3470
	(1,64)	0.0062	0.0008	0.4265	0.0809

**Table 3:** Parameter table.

**Block**	**Layer**	**#filters**	**Size**	**Activation**	**Options**
1	Conv2D	8	(1, 54)		Padding
2	DepthwiseConv2D	2^*^8	(8, 1)		
	Activation			ELU	
	AveragePool2D		(1, 4) for EEG+Hbo /(1, 16) for EEG+Hbr		
	Dropout				*P =* 0.2
3	SeparableConv2D	2^*^8	(1, 8)		padding
	Activation			ELU	
	AveragePool2D		(1, 16)for EEG+Hbo /(1, 12)for EEG+Hbr		
	Dropout				*P =* 0.2

### 3.4. Ablation analysis

The proposed fusion network architecture consisted of a temporal convolution layer, spatial convolution layer, and separable convolution layer, where the temporal convolution layer learned the time-frequency feature of each channel, the spatial convolution layer selected and extracted the spatial pattern of interesting channels, and separable convolution layer extracted global joint features and facilitated the design of a relatively lightweight network for small datasets. Feature fusion was conducted in these three modules, through which early fusion, middle fusion, and late fusion were configured and investigated. Ablation analysis was conducted to further explore the significance of multimodal fusion. We conducted the ablation experiments based on training strategy A and training strategy B, respectively.

**Figure 8:**
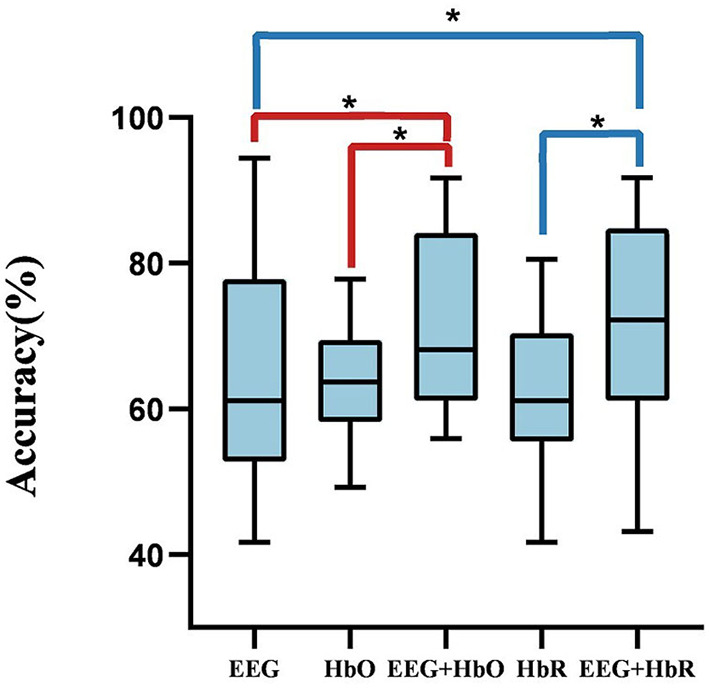
The comparison between unimodality and bimodality. A *P-*value of < 0.05 and the performance of bimodal fusion is significantly superior to that of unimodality.

First, we optimized the proposed bimodal fusion network to achieve the best classification performance. For training strategy A, we can conclude that, when using EEG-only, the average accuracy of all participants was 65.00% and the standard deviation was 2.11% using EEGNet with the same hyperparameters related to EEG in the bimodal process. When using HbO-fNIRS, the average accuracy was 63.13% and the standard deviation was 0.57% using ANN (consists of spatial convolution layer and separable convolution layer) with the same hyperparameters related to HbO in bimodal process. When using HbR-fNIRS, the average accuracy was 62.43% and the standard deviation was 1.08% using ANN with the same hyperparameters related to HbR in a bimodal process. The average accuracy was 71.60% using EEG-HbO with E-N-0. The average accuracy was 71.21% using EEG-HbR with E-N-0. Statistical analysis was performed by using the Wilcoxon signed-rank test to compare the performance of unimodality with that of bimodality. As shown in Figure 8, it was found that *P-*values were below 0.05, and a *P-*value of below 0.05 was regarded as statistically significant. The results are summarized in Figure 8, which demonstrated a consistent and significant model performance improvement, with the introduction of the other modalities. Multimodal fusion can complement advantages of each modality and improve classification performance significantly.

### 3.5. Voting results

**Figure 9:**
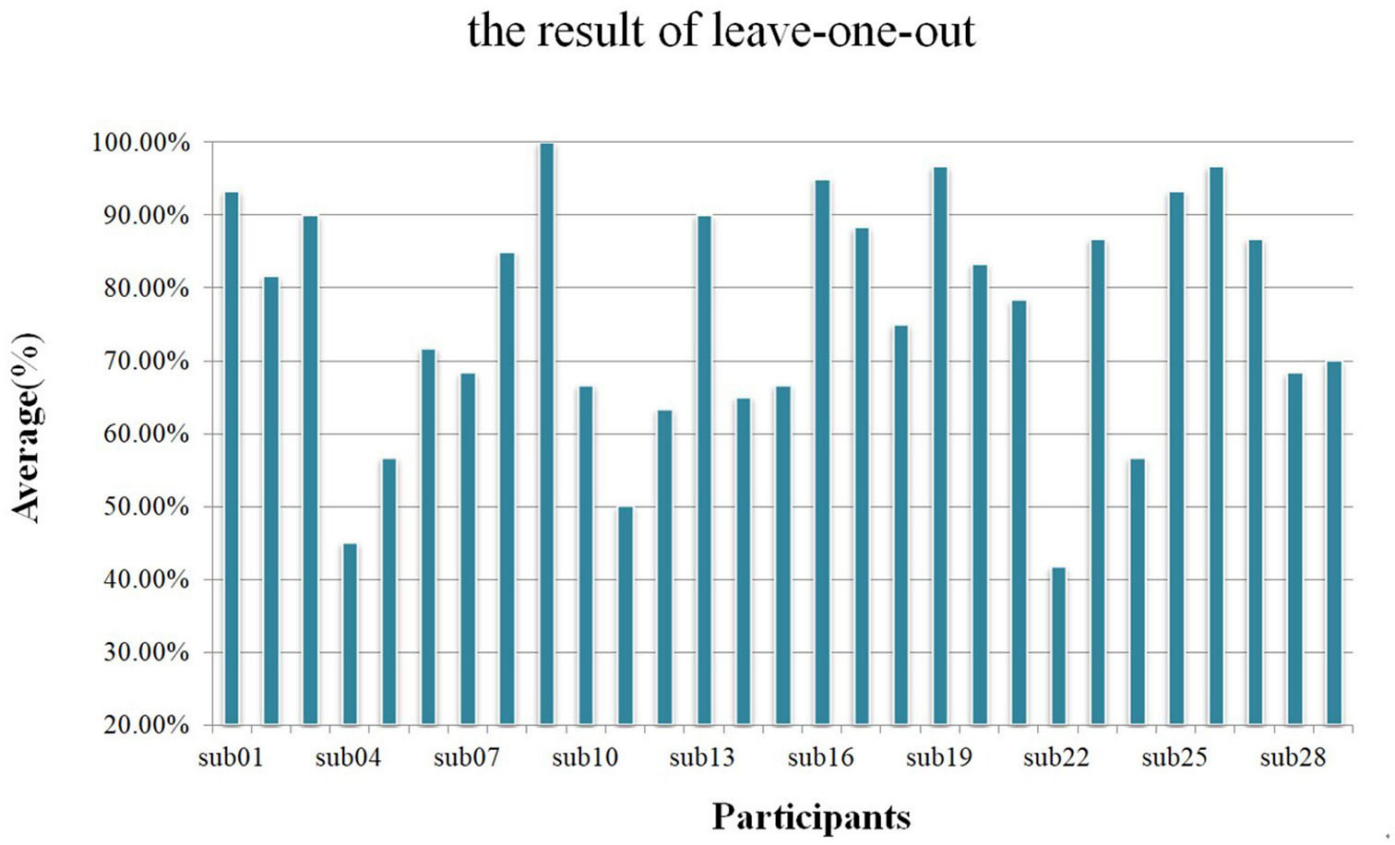
Using a leave-one-out analysis scheme, the average accuracy of each participant with the voting mechanism.

In this study, we divided each trial into 8 overlapping sub-trials and used the majority voting method to achieve the final result of each trial. We used training strategy B to train networks and used the same hyperparameter to perform 500 epochs. As shown in Figure 9, during the leave-one-out analysis, the average accuracy without the voting mechanism was 72.13% and the standard deviation was 0.1391, while the average accuracy with the voting mechanism was 76.21% and the standard deviation was 0.1611.

## 4. Discussion

Bimodal fusion methods demonstrated higher performance than that of unimodal data, whether using traditional machine learning methods with feature extraction classification schemes or deep learning methods with an end-to-end learning process. The heterogeneity between EEG and fNIRS data does exist; however, the heterogeneity is not as high as we thought based on the signal sources. In addition, incorporating special methods for bimodal fusion boosts BCI performance.

The classification results of the proposed Y-shape model are summarized in Figure 7, which contains conditions with different types of fNIRS data (Hbo vs. Hbr) and different kernel sizes on the first layers of the EEG branch. Figure 7A showed the fusion of EEG and Hbo, and Figure 7B showed the fusion of EEG and Hbr. According to the consistent performance of the two types of fNIRS information, models with an early fusion of EEG and fNIRS data have better classification accuracy than those of other stages, regardless of the size of the kernel and fNIRS data type. Comparing two types of fNIRS data, models with Hbo as input demonstrated higher resilience of model hyperparameter than the models using Hbr as input, although these two types of information were inherently correlated by the mechanism of blood supply in the human brain. Hbr data might be more sensitive to subtle changes in brain activities, which introduced more irrelevant activities other than motor imagery.

The common analysis for fNIRS signals was limited to the temporal-domain features such as the mean value, slope, peak, and so on, due to the low sampling rate. However, these features might not be informative enough to reflect the overall and detailed characteristics of fNIRS signals. Thus, the resultant information loss deteriorated the classification performance. We noticed that the classification accuracy of each participant with temporal convolution was lower than that without temporal convolution in fNIRS models using deep learning methods, which is consistent with our prior knowledge of fNIRS signals. In addition, the classification accuracies using deep learning methods for fNIRS signals (HbO-only, 63.13%) were better than that using traditional machine learning with handcrafted features and a predefined learning model (HbO-only, 58.33%), which demonstrated the superiority of the deep learning methods in the field of BCI research.

The average accuracy for the EEG-only model was 66.09% using traditional machine learning techniques, and for the HbO-only model, the average accuracy was 54.31% without data augmentation. With data augmentation, the highest average accuracy for EEG-only was 69.25%, and for the HbO-only model, the highest average accuracy was 58.33%. With data augmentation combined with deep learning methods, the highest average accuracy for the EEG-only model was 65.00%, and the HbO-only model was 63.13%. Therefore, the size of the dataset had a great impact on the classification performance of left-vs.-right MI tasks. An effective data augmentation method was able to boost model performance and improve generalization. The data augmentation method we propose in this study is valid and effective, especially for long recording interval paradigms when integrating EEG and fNIRS data.

Based on the classification results from different networks, it was clear that the early fusion techniques demonstrated significant positive impacts on the bimodal MI classification. A slight decreasing trend was observed with early, middle, and late fusion methods, respectively (shown in Figure 7 and Table 2). Although all three of these networks were feature-level fusion, the difference in model performance might be a compound effect of the heterogeneity of data, the level of feature (high-level features vs. low-level features), and bimodal co-adapted learning. Early stage fusion of bimodal data might have added additional constraints on the learning process and subsequently regularized the two feature extraction branches in the Y-shaped network. It seemed that early fusion could mitigate the loss of information. In previous studies in computer vision, it was suggested that multimodal data with higher heterogeneity tend to have better performance in late-fusion models, while multimodal data with low heterogeneity tend to perform better in the early fusion in the field of medical image (Ramachandram and Taylor, 2017; Mogadala et al., 2021; Yan et al., 2021). The heterogeneity of EEG and fNIRS might not be as high as we expected since they were able to be fused in the temporal domain, although these two types of data were recorded from completely different signal sources. However, this phenomenon was preliminarily observed and validated with only one open dataset due to limited access to bimodal BCI datasets of EEG and fNIRS; further analysis with more datasets should be done in the future. In addition, it was interesting that no statistically significant difference was found between middle-stage fusion and late-stage fusion, which might be caused by insufficient complementary features. The temporal-spatial feature of EEG and the spatial feature of fNIRS were extremely important.

In the dataset investigated in this study (Shin et al., 2017), there were only 30 trials for each task of each participant. The major limitation in the amount of data severely limited the scale of the neural network as well as the final classification performance. Future studies should be done to validate the conclusions in this study with a large bimodal EEG-fNIRS dataset. In addition, compared to the classification results in the literature, the proposed framework showed the same level of performance compared to that of the state-of-the-art methods (ours at 76.21 vs. 78.59% in the literature) (Kwak et al., 2022). More advanced learning techniques should be investigated to further improve the performance of the proposed network.

## 5. Conclusion

In this study, bimodal fusion methods of EEG and fNIRS were investigated with an open dataset. Compact Y-shaped ANN architectures are proposed and validated to investigate EEG-fNIRS fusion methods and strategies. The main framework of EEGNet is used in the proposed network. The results suggested that networks with EEG-fNIRS features integrated at an early stage demonstrated statistically higher accuracy compared to the other fusion methods in motor imagery classification tasks, which partially suggested that the heterogeneity of EEG and fNIRS might be relatively low despite the fact that these two types of signals were acquired from different sources. With the proposed framework, the final classification accuracy of the proposed method reached 76.21%, which was at the same level compared to the state-of-the-art on an EEG-fNIRS hybrid BCI open dataset in discriminating left and right motor imagery.

## Data availability statement

Publicly available datasets were analyzed in this study. This data can be found here: 10.1109/TNSRE.2016.2628057.

## Ethics statement

Ethical review and approval was not required for the study on human participants in accordance with the local legislation and institutional requirements. The patients/participants provided their written informed consent to participate in this study.

## Author contributions

YL conceived and designed the experiments, analyzed the experimental data, and wrote the manuscript. XZ conceived the experiments, guided the experiments, and participated in this study in the process of manuscript drafting and revision. DM gave some valuable suggestions and participated in this study as a consultant in the process of manuscript revision. All authors contributed to the article and approved the submitted version.
